# Supplementary material for: Gli1-expressing stromal cells are highly reparative precursors of long-lived chondroprogenitors in the fetal murine limb
Source: Nat Commun. 2025 Nov 18;16:10107. doi: 10.1038/s41467-025-65029-y (PMC12627582; doi:10.1038/s41467-025-65029-y)
Supplement: Supplementary file 1 — Supplementary Information [file 41467_2025_65029_MOESM1_ESM.pdf]

Supplementary Information

Gli1-expressing stromal cells are highly reparative precursors of long-lived chondroprogenitors in the fetal murine limb

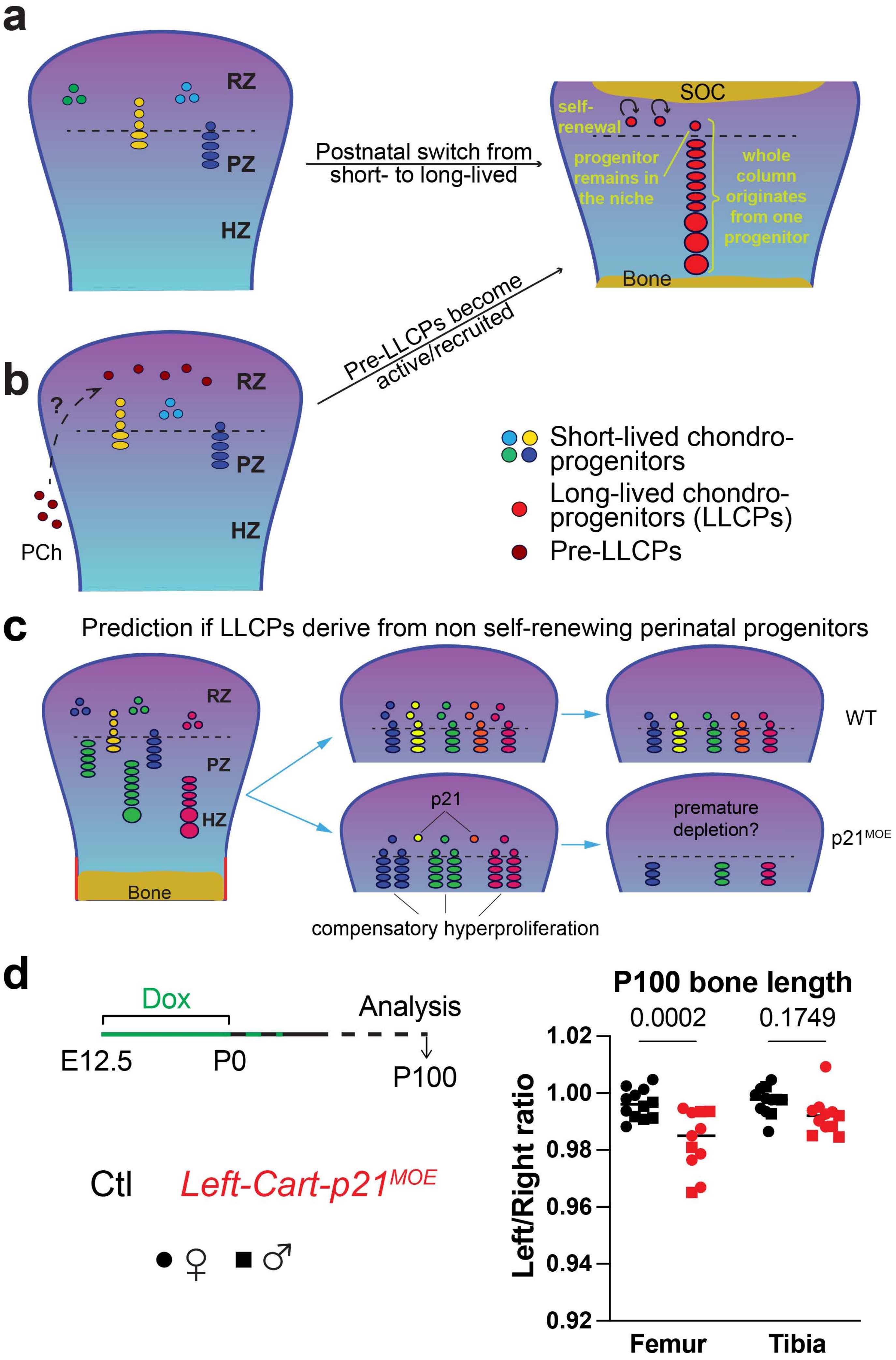

a
RZ
PZ
HZ
Postnatal switch from
short- to long-lived
SOC
self-
renewal
progenitor
remains in
the niche
whole
column
originates
from one
progenitor
Bone
Pre-LLCPs become
active/recruited
b
RZ
?
PZ
HZ
PCh
Short-lived chondro-
progenitors
Long-lived chondro-
progenitors (LLCPs)
Pre-LLCPs
c
Prediction if LLCPs derive from non self-renewing perinatal progenitors
RZ
PZ
HZ
Bone
WT
p21
premature
depletion?
p21MOE
compensatory hyperproliferation
d
Dox
Analysis
E12.5
P0
P100
Ctl
Left-Cart-p21MOE
P100 bone length
0.0002
0.1749
1.02
1.00
0.98
0.96
0.94
0.92
Left/Right ratio
Femur
Tibia

Supplementary Figure 1. Possible origins of postnatal cartilage progenitors (CPs). **a**, Cartilage-resident short-lived CPs eventually become long-lived, due to intrinsic and/or extrinsic triggers. **b**, Precursors of long-lived CPs (Pre-LLCPs) already exist in the fetal limb as a separate population (in the cartilage and/or outside), and only become active/recruited postnatally, in response to cartilage maturation. RZ, PZ, HZ, resting, proliferative, hypertrophic zones; SOC, secondary ossification centre; PCh, perichondrium. **c**, Expected accelerated depletion of chondroprogenitors in response to mosaic p21 expression. **d**, Minor to no asymmetries were generated by P100 despite the overactivation of perinatal CPs.

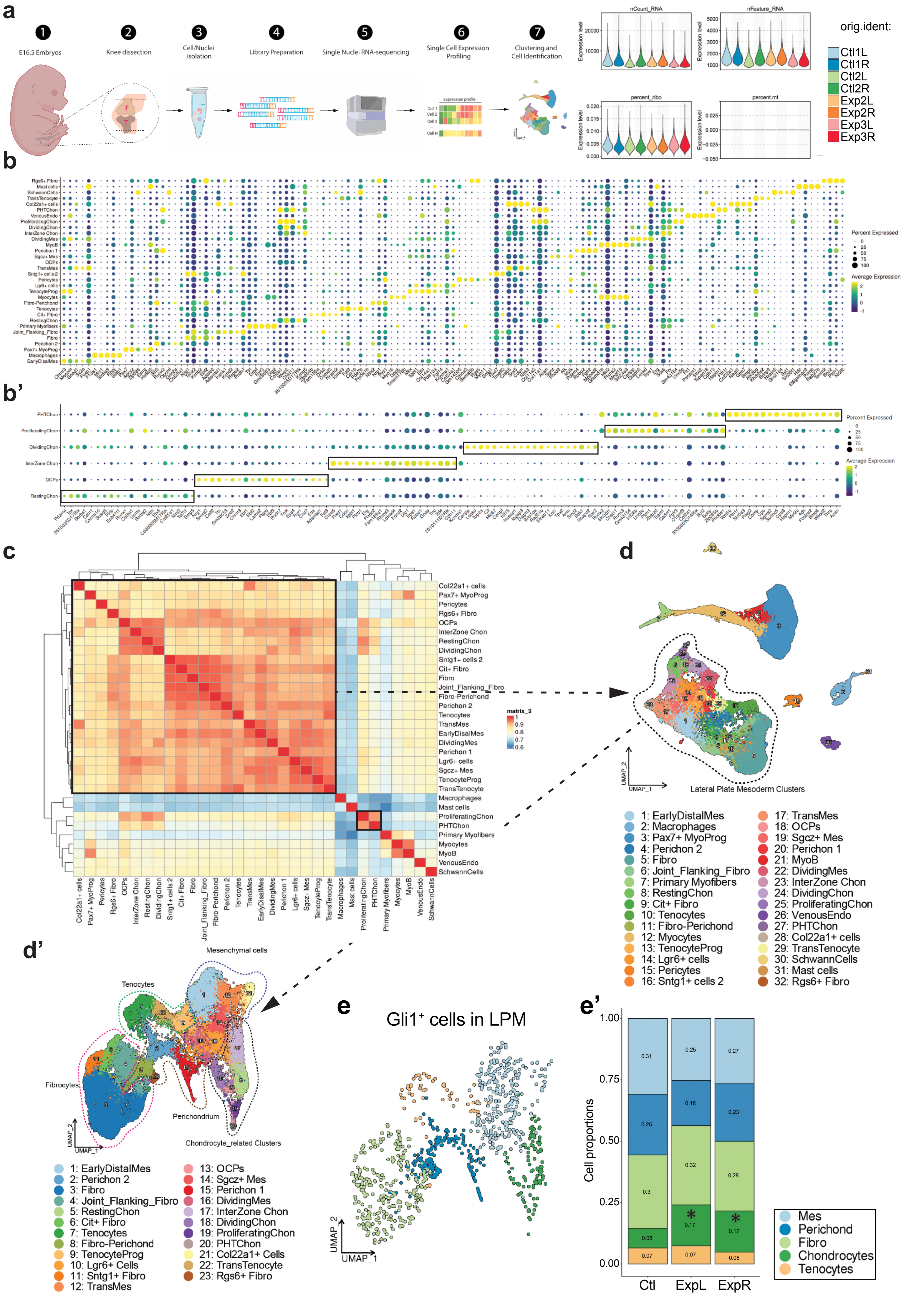

a
E16.5 Embryos
Knee dissection
Cell/Nuclei isolation
Library Preparation
Single Nuclei RNA-sequencing
Single Cell Expression Profiling
Clustering and Cell Identification
nCount_RNA
nFeature_RNA
percent_ribo
percent.mt
Expression level
orig.ident:
Ctl1L
Ctl1R
Ctl2L
Ctl2R
Exp2L
Exp2R
Exp3L
Exp3R
b
Percent Expressed
Average Expression
b'
PHTChon
ProliferatingChon
DividingChon
InterZone Chon
OCPs
RestingChon
c
matrix_3
Col22a1+ cells
Pax7+ MyoProg
Pericytes
Rgs6+ Fibro
OCPs
InterZone Chon
RestingChon
DividingChon
Sntg1+ cells 2
Cit+ Fibro
Fibro
Joint_Flanking_Fibro
Fibro-Perichond
Perichon 2
Tenocytes
TransMes
EarlyDisalMes
DividingMes
Perichon 1
Lgr6+ cells
Sgcz+ Mes
TenocyteProg
TransTenocyte
Macrophages
Mast cells
ProliferatingChon
PHTChon
Primary Myofibers
Myocytes
MyoB
VenousEndo
SchwannCells
d
Lateral Plate Mesoderm Clusters
UMAP_1
UMAP_2
1: EarlyDistalMes
2: Macrophages
3: Pax7+ MyoProg
4: Perichon 2
5: Fibro
6: Joint_Flanking_Fibro
7: Primary Myofibers
8: RestingChon
9: Cit+ Fibro
10: Tenocytes
11: Fibro-Perichond
12: Myocytes
13: TenocyteProg
14: Lgr6+ cells
15: Pericytes
16: Sntg1+ cells 2
17: TransMes
18: OCPs
19: Sgcz+ Mes
20: Perichon 1
21: MyoB
22: DividingMes
23: InterZone Chon
24: DividingChon
25: ProliferatingChon
26: VenousEndo
27: PHTChon
28: Col22a1+ cells
29: TransTenocyte
30: SchwannCells
31: Mast cells
32: Rgs6+ Fibro
d'
Mesenchymal cells
Tenocytes
Fibrocytes
Perichondrium
Chondrocyte_related Clusters
1: EarlyDistalMes
2: Perichon 2
3: Fibro
4: Joint_Flanking_Fibro
5: RestingChon
6: Cit+ Fibro
7: Tenocytes
8: Fibro-Perichond
9: TenocyteProg
10: Lgr6+ Cells
11: Sntg1+ Fibro
12: TransMes
13: OCPs
14: Sgcz+ Mes
15: Perichon 1
16: DividingMes
17: InterZone Chon
18: DividingChon
19: ProliferatingChon
20: PHTChon
21: Col22a1+ Cells
22: TransTenocyte
23: Rgs6+ Fibro
e
Gli1+ cells in LPM
UMAP_1
UMAP_2
e'
Cell proportions
Ctl
ExpL
ExpR
Mes
Perichon
Fibro
Chondrocytes
Tenocytes

Supplementary Figure 2. Analysis of snRNA-seq data. **a**, Schematic of the process for sample collection, processing of single-nuclei RNA sequencing and quality control in Seurat processing. **b-b'**, Dotplot graph illustrating the markers of each of the 32 initially identified clusters (b) and chondrocyte-related clusters only (b'). **c**, Heatmap of average gene expression of all clusters showing the accuracy of clustering. **d-d'**, UMAP of all identified clusters (d). The highly correlated clusters (lateral plate mesoderm-derived cells, LPM) are shown boxed by black rectangles on the heatmap (c), and subclustered in (d'). **e-e'**, Gli1^{+} cells in the LPM group (e) and distribution of LPM populations within Gli1^{+} cells in Ctl and Exp limbs (e'). * denotes a significant difference identified by Propeller.

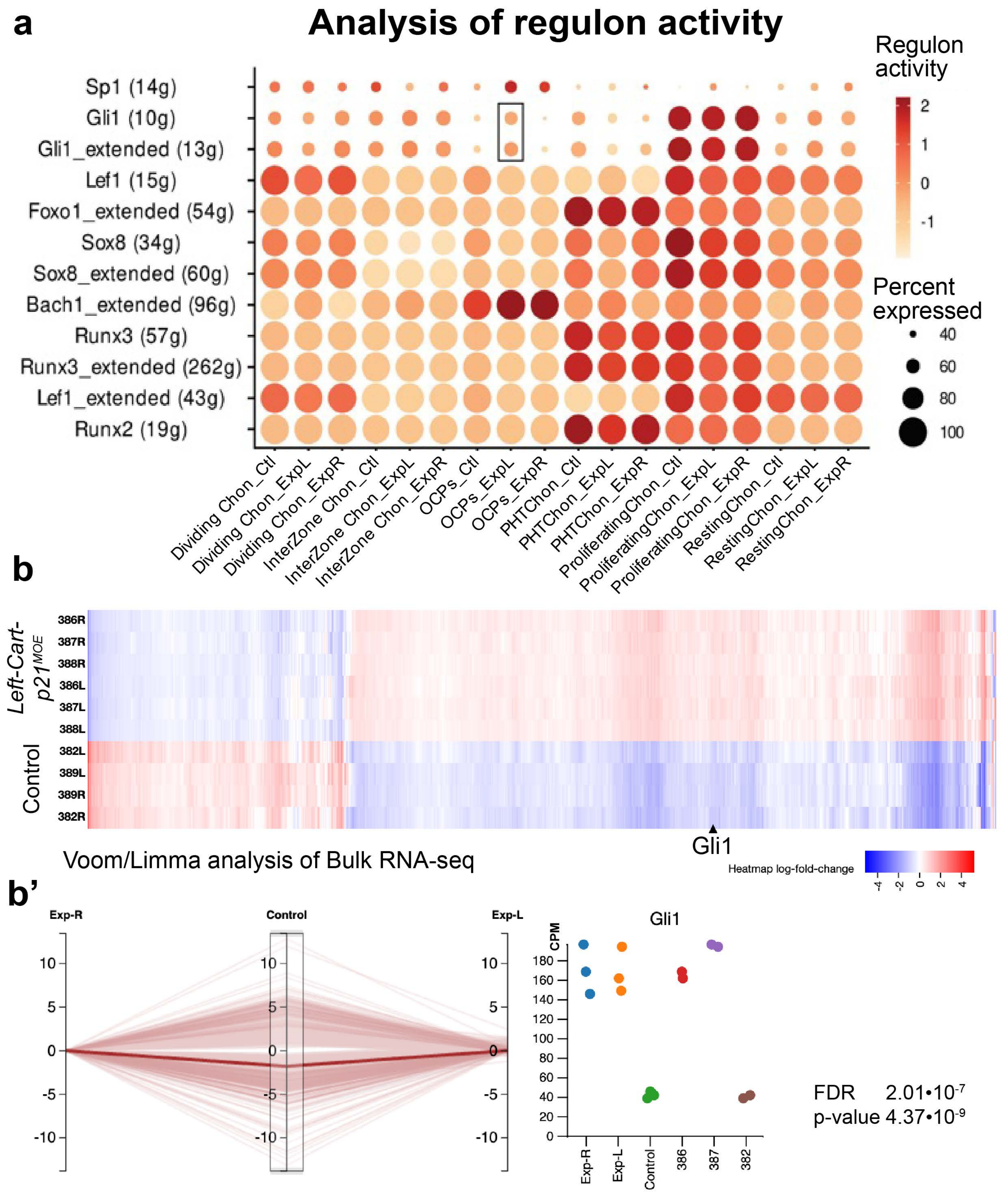
a
Analysis of regulon activity
Sp1 (14g)
Gli1 (10g)
Gli1_extended (13g)
Lef1 (15g)
Foxo1_extended (54g)
Sox8 (34g)
Sox8_extended (60g)
Bach1_extended (96g)
Runx3 (57g)
Runx3_extended (262g)
Lef1_extended (43g)
Runx2 (19g)
Regulon activity
2
1
0
-1
Percent expressed
40
60
80
100
Dividing Chon_Ctl
Dividing Chon_ExpL
Dividing Chon_ExpR
InterZone Chon_Ctl
InterZone Chon_ExpL
InterZone Chon_ExpR
OCPs_Ctl
OCPs_ExpL
OCPs_ExpR
PHTChon_Ctl
PHTChon_ExpL
PHTChon_ExpR
ProliferatingChon_Ctl
ProliferatingChon_ExpL
ProliferatingChon_ExpR
RestingChon_Ctl
RestingChon_ExpL
RestingChon_ExpR
b
Left-Cart-p21^MOE
386R
387R
388R
386L
387L
388L
Control
382L
389L
389R
382R
Gli1
Voom/Limma analysis of Bulk RNA-seq
Heatmap log-fold-change
-4
-2
0
2
4
b'
Exp-R
Control
Exp-L
10
5
0
-5
-10
Gli1
CPM
180
160
140
120
100
80
60
40
20
0
Exp-R
Exp-L
Control
386
387
382
FDR 2.01•10^-7
p-value 4.37•10^-9

Supplementary Figure 3. Combined SCENIC and bulk RNA-seq analysis. **a**, Chondrocyte-related cells were analysed via SCENIC and relevant regulons are shown. **b**, **b'**, Bulk RNA-seq data of left and right E17.5 cartilage from *Left-Cart-p21*^{MOE} (386, 387, 388) and Control embryos (382, 389) was analysed by Voom/Limma using *Degust*[1] (b, FDR threshold 0.05 and logFC threshold 1). Among the regulons shown in (a), the expression of *Gli1* was found differentially expressed (b', CPM: counts per million bp).

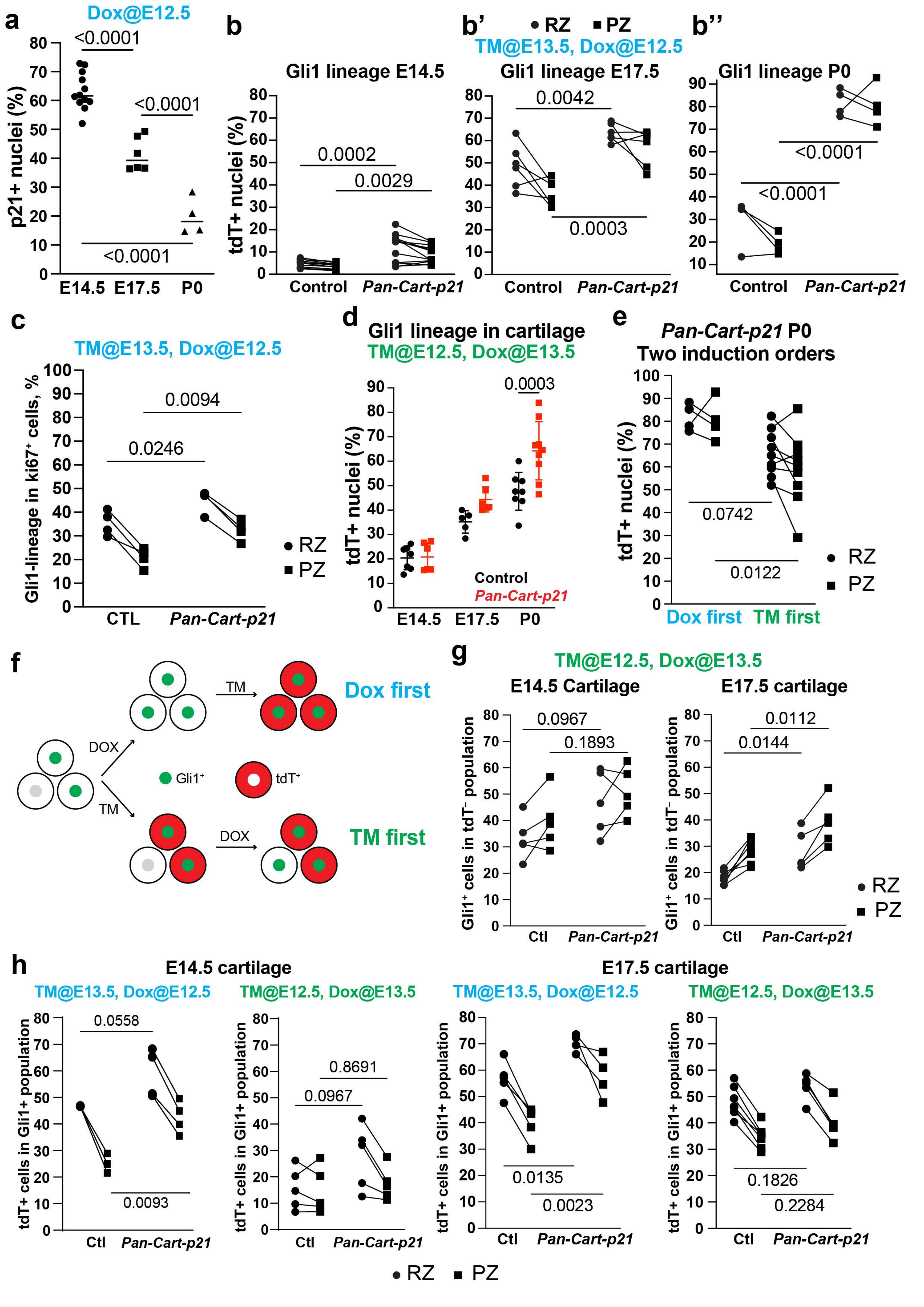
a
Dox@E12.5
p21+ nuclei (%)
<0.0001
<0.0001
<0.0001
E14.5 E17.5 P0
b
Gli1 lineage E14.5
tdT+ nuclei (%)
0.0002
0.0029
Control Pan-Cart-p21
b'
• RZ ▪ PZ
TM@E13.5, Dox@E12.5
Gli1 lineage E17.5
0.0042
0.0003
Control Pan-Cart-p21
b''
Gli1 lineage P0
<0.0001
<0.0001
Control Pan-Cart-p21
c
TM@E13.5, Dox@E12.5
Gli1-lineage in ki67+ cells, %
0.0094
0.0246
CTL Pan-Cart-p21
RZ
PZ
d
Gli1 lineage in cartilage
TM@E12.5, Dox@E13.5
tdT+ nuclei (%)
0.0003
Control
Pan-Cart-p21
E14.5 E17.5 P0
e
Pan-Cart-p21 P0
Two induction orders
tdT+ nuclei (%)
0.0742
0.0122
Dox first TM first
RZ
PZ
f
TM
Dox first
DOX
Gli1+
tdT+
TM
DOX
TM first
g
TM@E12.5, Dox@E13.5
E14.5 Cartilage
Gli1+ cells in tdT− population
0.0967
0.1893
Ctl Pan-Cart-p21
E17.5 cartilage
Gli1+ cells in tdT− population
0.0112
0.0144
Ctl Pan-Cart-p21
RZ
PZ
h
E14.5 cartilage
TM@E13.5, Dox@E12.5
tdT+ cells in Gli1+ population
0.0558
0.0093
Ctl Pan-Cart-p21
TM@E12.5, Dox@E13.5
tdT+ cells in Gli1+ population
0.8691
0.0967
Ctl Pan-Cart-p21
E17.5 cartilage
TM@E13.5, Dox@E12.5
tdT+ cells in Gli1+ population
0.0135
0.0023
Ctl Pan-Cart-p21
TM@E12.5, Dox@E13.5
tdT+ cells in Gli1+ population
0.1826
0.2284
Ctl Pan-Cart-p21
• RZ ▪ PZ

Supplementary Figure 4. Characterisation of Gli1-lineage and non-Gli1-lineage behaviours with different induction regimens. **a**, **a'**, % of $p21^+$ nuclei found in the proximal tibia cartilage of *Pan-Cart-p21* mice at the shown stages, with Dox at E12.5 (a) or E13.5 (a'). p-values of multiple-comparisons tests after ANOVA are shown. **b-b''**, The Gli1 lineage was quantified in resting (RZ) and proliferative zone (PZ) of Ctl and *Pan-Cart-p21* samples at E14.5 (b), E17.5 (b') and P0 (b''). **c**, Proportion of tdT^+ (left) or $tdT^–$ cells (right) within the $Ki67^+$ chondrocytes, in RZ and PZ. **d**, Distribution of tdT^+ cells in Ctl and *Pan-Cart-p21^{MOE}* samples across stages, with a TM-first approach. **e,** Distribution of tdT^+ cells in *Pan-Cart-p21^{MOE}* P0 samples, comparing Dox-first with TM-first approach. **f**, Quantification of the proportion of non-Gli1 lineage cells ($tdT^–$) that express Gli1 in the RZ and PZ of the TM-first condition, at the indicated stages. In b-f, p-values for multiple comparisons tests after 2-way ANOVA are shown.

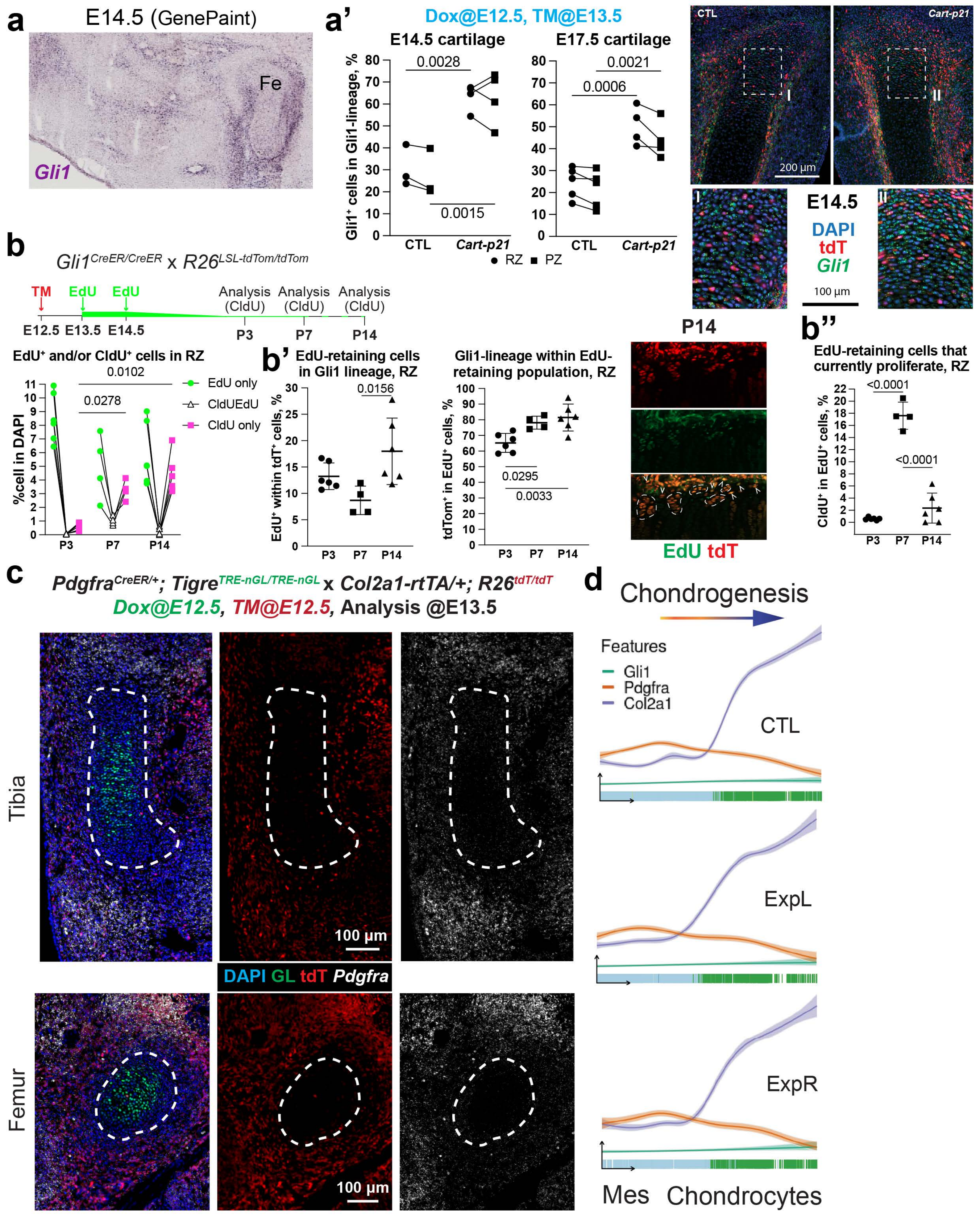

a
E14.5 (GenePaint)
Fe
Gli1
a'
Dox@E12.5, TM@E13.5
E14.5 cartilage
E17.5 cartilage
Gli1+ cells in Gli1-lineage, %
0.0028
0.0015
0.0021
0.0006
CTL
Cart-p21
● RZ ■ PZ
CTL
Cart-p21
200 µm
E14.5
DAPI
tdT
Gli1
100 µm
b
Gli1CreER/CreER x R26LSL-tdTom/tdTom
TM
EdU
EdU
Analysis (CldU)
Analysis (CldU)
Analysis (CldU)
E12.5 E13.5 E14.5
P3
P7
P14
EdU+ and/or CldU+ cells in RZ
%cell in DAPI
0.0102
0.0278
EdU only
CldUEdU
CldU only
b'
EdU-retaining cells in Gli1 lineage, RZ
EdU+ within tdT+ cells, %
0.0156
Gli1-lineage within EdU-retaining population, RZ
tdTom+ in EdU+ cells, %
0.0295
0.0033
P14
EdU tdT
b''
EdU-retaining cells that currently proliferate, RZ
CldU+ in EdU+ cells, %
<0.0001
<0.0001
c
PdgfraCreER/+; TigreTRE-nGL/TRE-nGL x Col2a1-rtTA/+; R26tdT/tdT
Dox@E12.5, TM@E12.5, Analysis @E13.5
Tibia
Femur
100 µm
DAPI GL tdT Pdgfra
100 µm
d
Chondrogenesis
Features
Gli1
Pdgfra
Col2a1
CTL
ExpL
ExpR
Mes
Chondrocytes

Supplementary Figure 5. Distribution and relationship of Gli1- and Pdgfra-expressing populations. **a-a'**, Expression of *Gli1* by ISH in the E14.5 hindlimb (a, image from GenePaint; Fe, femur, Ti, tibia) and quantification of *Gli1* within the E13.5-labelled Gli1 lineage (a', p-values for Sidak's multiple comparisons tests after 2-way ANOVA are shown). **b-b''** Experimental design (b, top) and quantification (b, bottom) of EdU^+ and/or $CldU^+$ cells, as well as EdU^+CldU^+/EdU^+ cells (b'') in the resting zone (RZ) at P3 (n=6), P7 (n=4) and P14 (n=6). Within the tdT population, we quantified the number of EdU^+ ones, and also tdT^+ ones within the EdU^+ population (b'). **c**, Localisation of Pdgfra-lineage (tdT, traced from E13.5 to E14.5) and *Pdgfra* mRNA is mostly located outside the cartilage elements (marked by GL expression). **d**, single-nuclei data was ordered from mesenchymal to chondrocyte fate (for Ctl, ExpL and ExpR). Expression of *Gli1*, *Pdgfra* and *Col2a1* is shown along this trajectory.

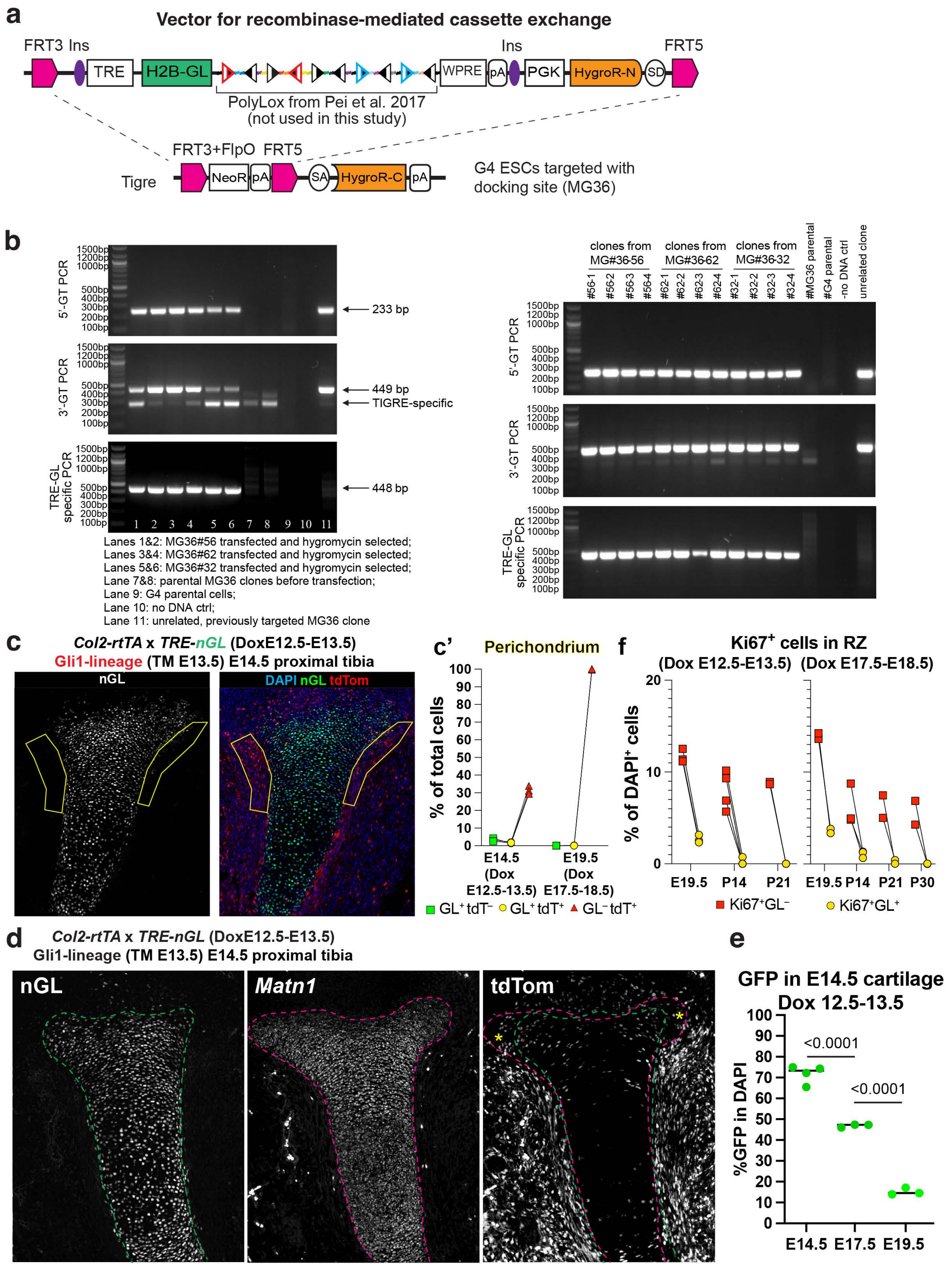

a
Vector for recombinase-mediated cassette exchange
FRT3 Ins
TRE
H2B-GL
WPRE
pA
Ins
PGK
HygroR-N
SD
FRT5
PolyLox from Pei et al. 2017
(not used in this study)
FRT3+FlpO FRT5
Tigre
NeoR
pA
SA
HygroR-C
pA
G4 ESCs targeted with
docking site (MG36)
b
5'-GT PCR
3'-GT PCR
TRE-GL specific PCR
233 bp
449 bp
TIGRE-specific
448 bp
Lanes 1&2: MG36#56 transfected and hygromycin selected;
Lanes 3&4: MG36#62 transfected and hygromycin selected;
Lanes 5&6: MG36#32 transfected and hygromycin selected;
Lane 7&8: parental MG36 clones before transfection;
Lane 9: G4 parental cells;
Lane 10: no DNA ctrl;
Lane 11: unrelated, previously targeted MG36 clone
clones from MG#36-56
clones from MG#36-62
clones from MG#36-32
#MG36 parental
#G4 parental
-no DNA ctrl
unrelated clone
c
Col2-rtTA x TRE-nGL (DoxE12.5-E13.5)
Gli1-lineage (TM E13.5) E14.5 proximal tibia
nGL
DAPI nGL tdTom
c'
Perichondrium
% of total cells
E14.5 (Dox E12.5-13.5)
E19.5 (Dox E17.5-18.5)
GL⁺ tdT⁻
GL⁺ tdT⁺
GL⁻ tdT⁺
f
Ki67⁺ cells in RZ
(Dox E12.5-E13.5)
(Dox E17.5-E18.5)
% of DAPI⁺ cells
E19.5
P14
P21
P30
Ki67⁺GL⁻
Ki67⁺GL⁺
d
Col2-rtTA x TRE-nGL (DoxE12.5-E13.5)
Gli1-lineage (TM E13.5) E14.5 proximal tibia
nGL
Matn1
tdTom
e
GFP in E14.5 cartilage
Dox 12.5-13.5
%GFP in DAPI
<0.0001
<0.0001
E14.5
E17.5
E19.5

Supplementary Figure 6. Generation and characterisation of new targeted cell and mouse lines for pulse-chase experiments. **a**, Targeting strategy to generate ES cell lines carrying a Tet-responsive element (TRE)-controlled nuclear green lantern (H2B-GL) gene in the Tigre locus. Successful FlpO-mediated cassette exchange reconstitutes the Hygro-resistance gene. SA, splice acceptor. pA, poly A. Ins, insulator. FRT, flippase recognition target. WPRE, RNA-stabilising sequence from Woodchuck virus. PGK, constitutive mammalian promoter. **b**, Confirmation of initially picked clones (left) and subclones (right) by 5'/3'/TRE-GL specific PCR reactions. **c**, **c'**, Representative images (c) and quantification in the yellow region of interest (c') of the single- and double-labelled cells, upon TM and Dox induction, as indicated. **d**, *Matn1* HCR was used to identify the cartilage, and by imaging tdTom and nGL at the same time, we found that there were barely any $tdTom^+$ chondrocytes that were not nGL^+ (except in the region indicated with an asterisk). Dashed lines delimit the nGL^+ (green) or the $Matn1^+$ (magenta) region. n=4. **e**, Quantification of nGL^+ cells in the cartilage at the indicated stages. **f**, Quantification of $Ki67^+$ (i.e., cycling) cells, both GL^+ and $GL^–$, in the RZ. Stages and Dox treatment are indicated.

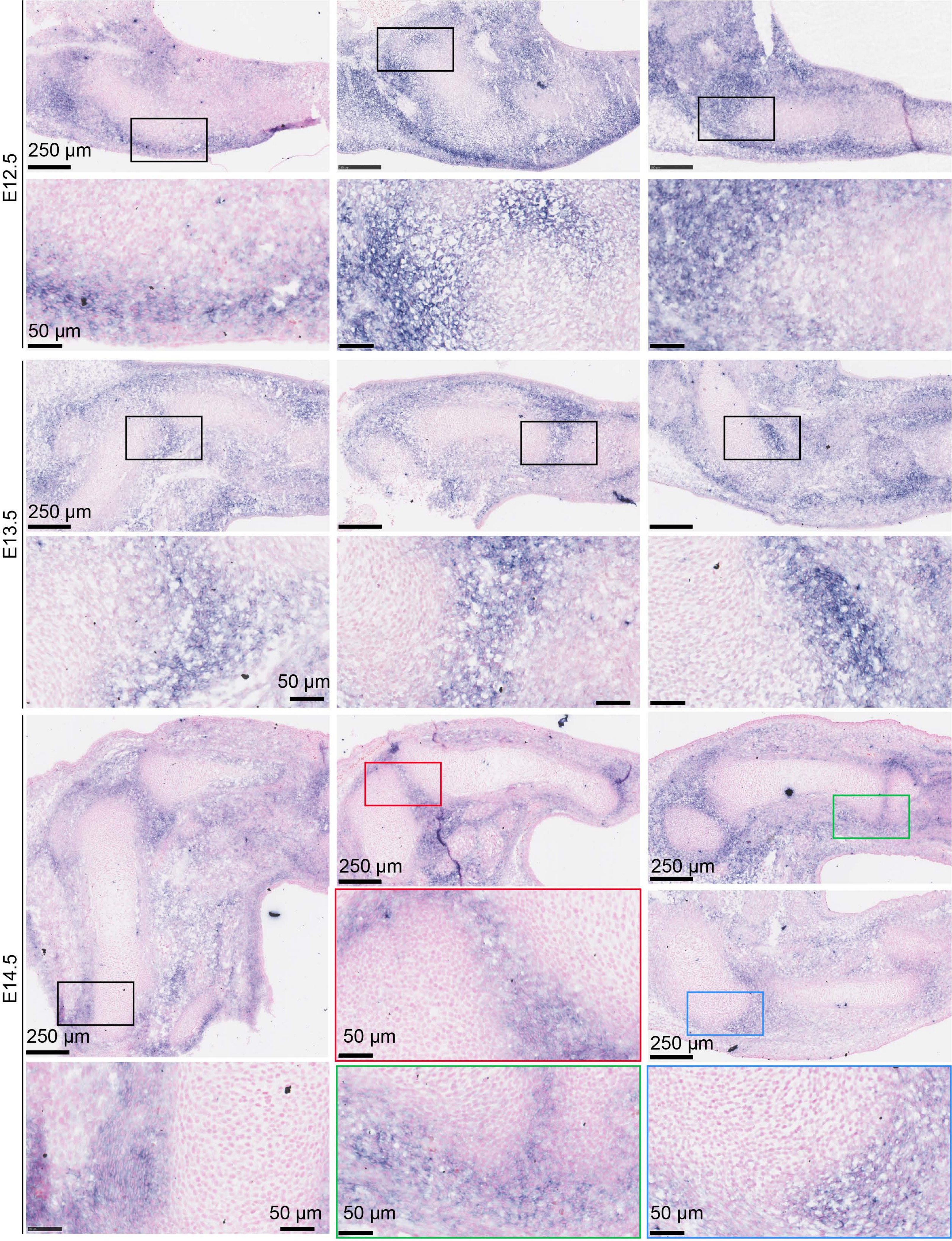

E12.5
E13.5
E14.5
250 µm
50 µm

Supplementary Figure 7. Expression of *Pdgfra* by ISH at the indicated stages. Rectangles denote regions that are shown magnified.

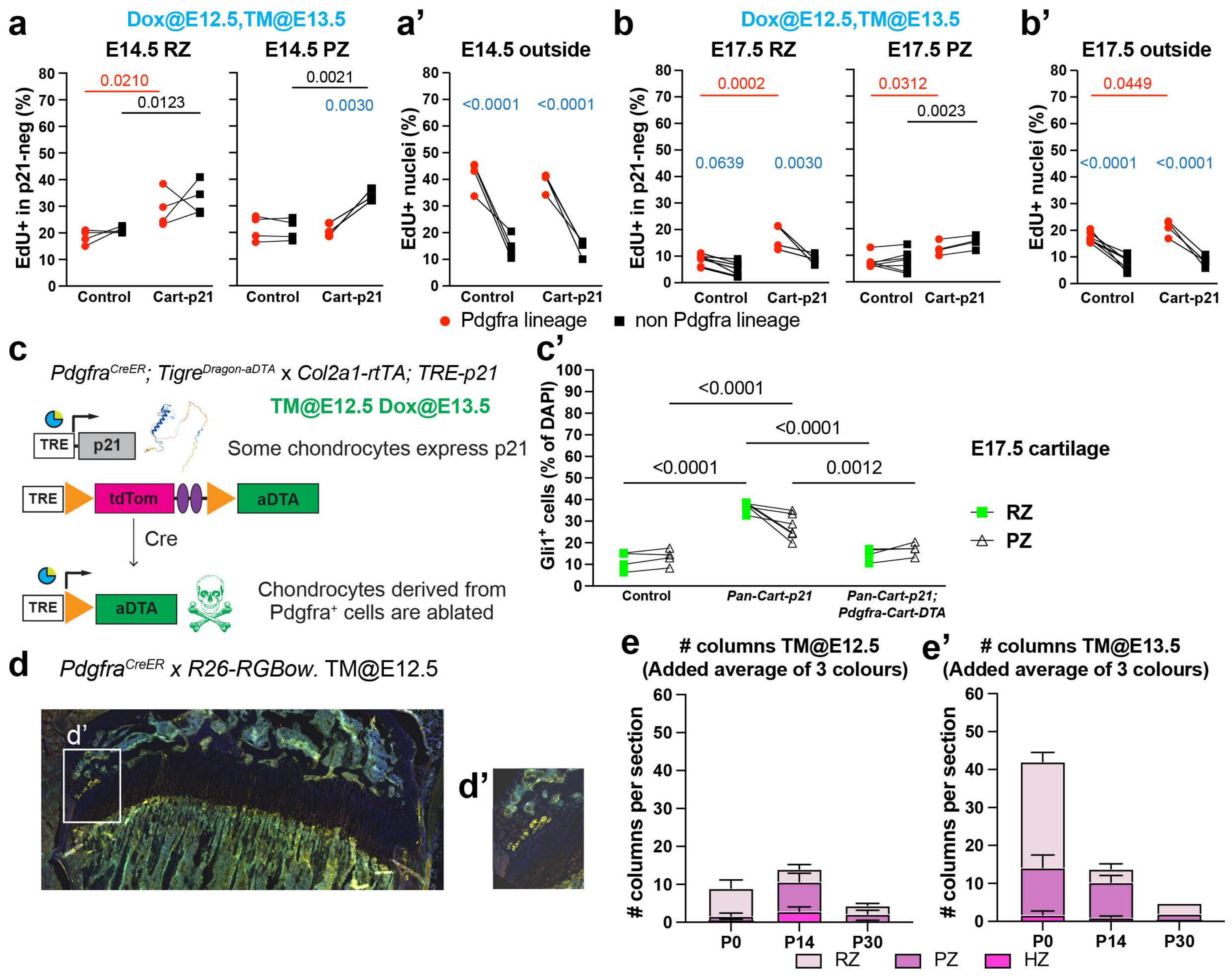

a
Dox@E12.5,TM@E13.5
E14.5 RZ
E14.5 PZ
EdU+ in p21-neg (%)
0.0210
0.0123
0.0021
0.0030
Control
Cart-p21
a'
E14.5 outside
EdU+ nuclei (%)
<0.0001
<0.0001
b
Dox@E12.5,TM@E13.5
E17.5 RZ
E17.5 PZ
0.0002
0.0639
0.0030
0.0312
0.0023
b'
E17.5 outside
0.0449
<0.0001
<0.0001
Pdgfra lineage
non Pdgfra lineage
c
PdgfraCreER; TigreDragon-aDTA x Col2a1-rtTA; TRE-p21
TM@E12.5 Dox@E13.5
TRE
p21
Some chondrocytes express p21
tdTom
aDTA
Cre
Chondrocytes derived from Pdgfra+ cells are ablated
c'
Gli1+ cells (% of DAPI)
<0.0001
<0.0001
<0.0001
0.0012
Control
Pan-Cart-p21
Pan-Cart-p21; Pdgfra-Cart-DTA
E17.5 cartilage
RZ
PZ
d
PdgfraCreER x R26-RGBow. TM@E12.5
d'
e
columns TM@E12.5
(Added average of 3 colours)
e'
columns TM@E13.5
(Added average of 3 colours)
columns per section
P0
P14
P30
RZ
PZ
HZ

Supplementary Figure 8. Characterisation and manipulation of the Pdgfra lineage. **a**-**b'**, Quantification of EdU in p21-negative chondrocytes of resting and proliferative zones (a, b, as indicated) and in cells outside the cartilage (a', b'), at E14.5 (a-a') and E17.5 (b-b'), distinguishing between Pdgfra-lineage and non-lineage cells. In (a-b'), p-values for multiple comparisons test after 2-way ANOVA are shown. **c-c'**, Ablation of Pdgfra-derived chondrocytes in the context of *Pan-Cart-p21*. Crosses and transgene induction as shown in c. Quantification of $Gli1^{+}$ cells by HCR is shown in c' (n=4 Control, 6 *Pan-Cart-p21* and 3 *Pan-Cart-p21; Pdgfra-Cart-DTA*). **d-e'**, Tri-colour lineage tracing of $Pdgfra^{+}$ cells, upon TM injection at E12.5 (d, e, boxed region amplified in d') and E13.5 (e'). For the quantifications in d and d', the average number of columns of each colour per sections was added up to obtain a total number of columns per section per sample.

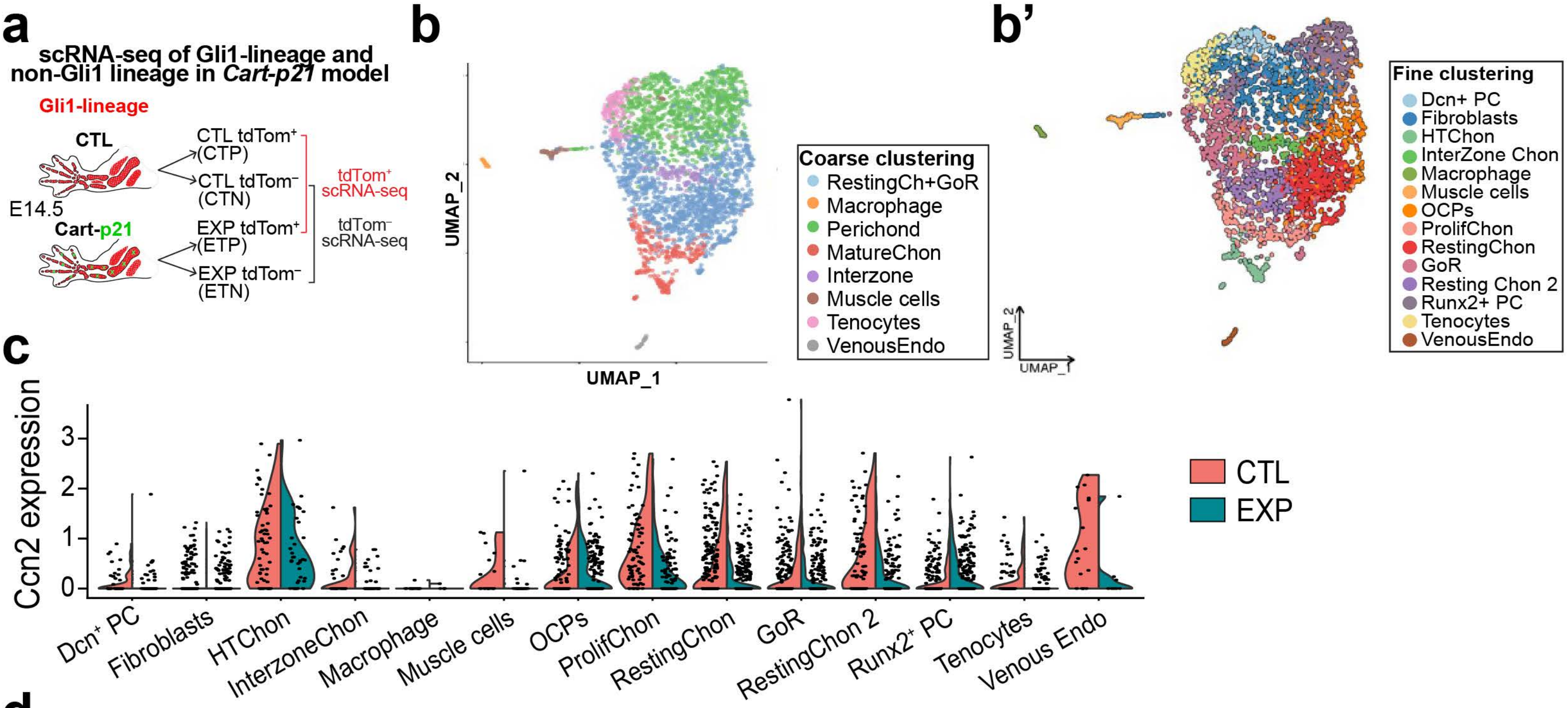

d

GO terms related to *Ccn2* (EXP vs. CTL)

Pathway	NES	pval	padj
GOBP_CELLULAR_RESPONSE_TO_STRESS	-1.31	7.8e-04	3.1e-02
GOBP_CELL_CYCLE	-1.46	2.5e-04	2.2e-02
GOBP_CELL_CYCLE_PROCESS	-1.52	2.5e-04	2.2e-02
GOBP_REGULATION_OF_CELL_CYCLE	-1.55	2.4e-04	2.2e-02
GOBP_POSITIVE_REGULATION_OF_CELL_CYCLE	-1.56	6.8e-04	3.0e-02
GOBP_SKELETAL_SYSTEM_MORPHOGENESIS	-1.63	8.9e-04	3.4e-02
GOBP_REGULATION_OF_CELL_CYCLE_PROCESS	-1.67	2.3e-04	2.2e-02
GOBP_SKELETAL_SYSTEM_DEVELOPMENT	-1.67	2.3e-04	2.2e-02
GOBP_DNA_METABOLIC_PROCESS	-1.68	2.4e-04	2.2e-02
GOCC_EXTERNAL_ENCAPSULATING_STRUCTURE	-1.70	2.2e-04	2.2e-02
GOBP_EXTRACELLULAR_STRUCTURE_ORGANIZATION	-1.75	2.3e-04	2.2e-02
GOBP_CONNECTIVE_TISSUE_DEVELOPMENT	-1.93	2.3e-04	2.2e-02
GOBP_CHONDROCYTE_DIFFERENTIATION	-1.96	2.2e-04	2.2e-02
GOBP_CARTILAGE_DEVELOPMENT	-2.11	2.2e-04	2.2e-02

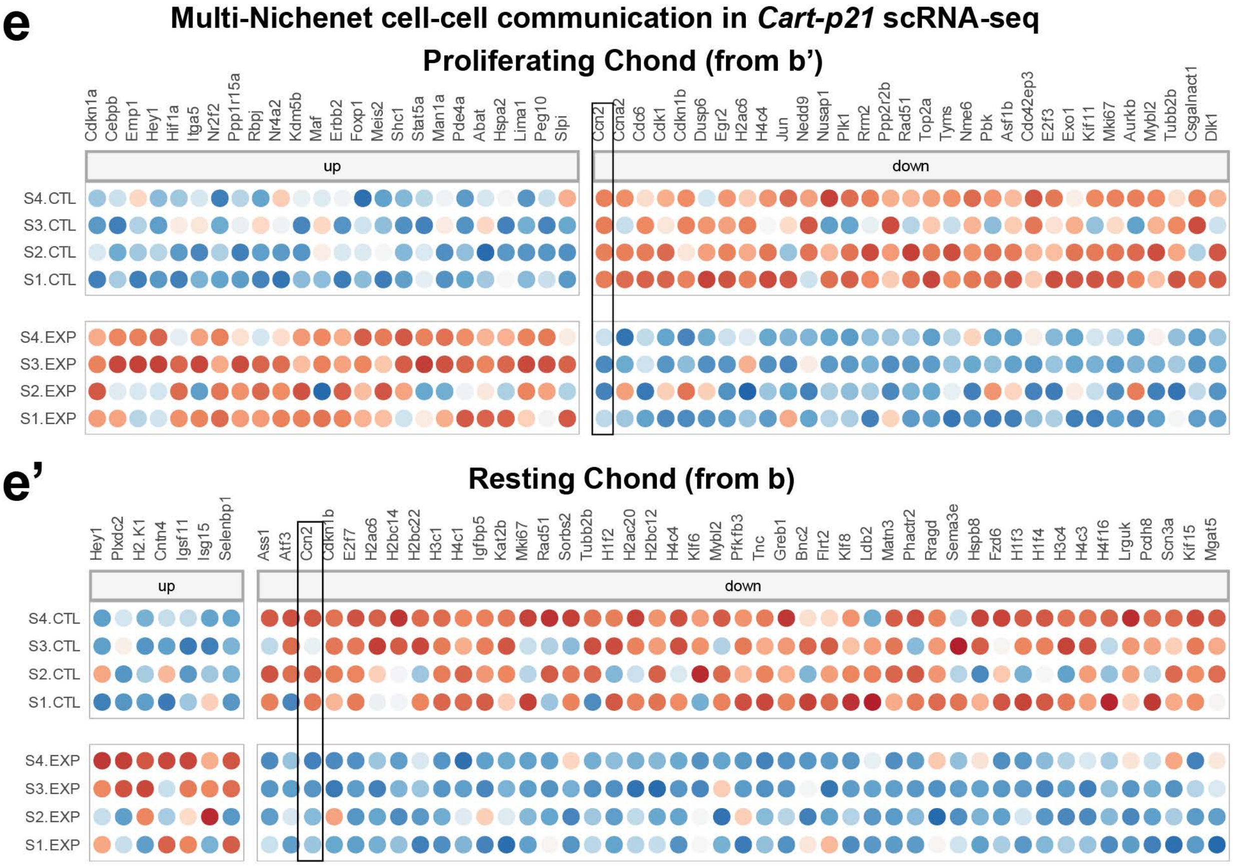

Supplementary Figure 9. scRNA-seq analysis reveals cell-cell communication via CCN2. **a**, Design of the lineage-specific single-cell RNA-seq approach, multiplexed by sex. **b**, **b'**, Coarse (b) and fine (b') clustering outcome of the curated sequencing data (see Online Methods). **c**, Violin plot showing expression of *Ccn2* in the different populations identified in the scRNA-seq data, for the 2 genotypes. CTL= CTN+CTP, EXP= ETN+ETP. **d**, Gene Ontology terms related to Ccn2, differentially enriched in EXP vs. CTL samples. **e, e'**, MultiNicheNet analysis of cell-cell communication in Proliferating Chondrocytes (e), as well as Resting chondrocytes (e') of the scRNA-seq dataset. In e, e', Ccn2 is boxed.

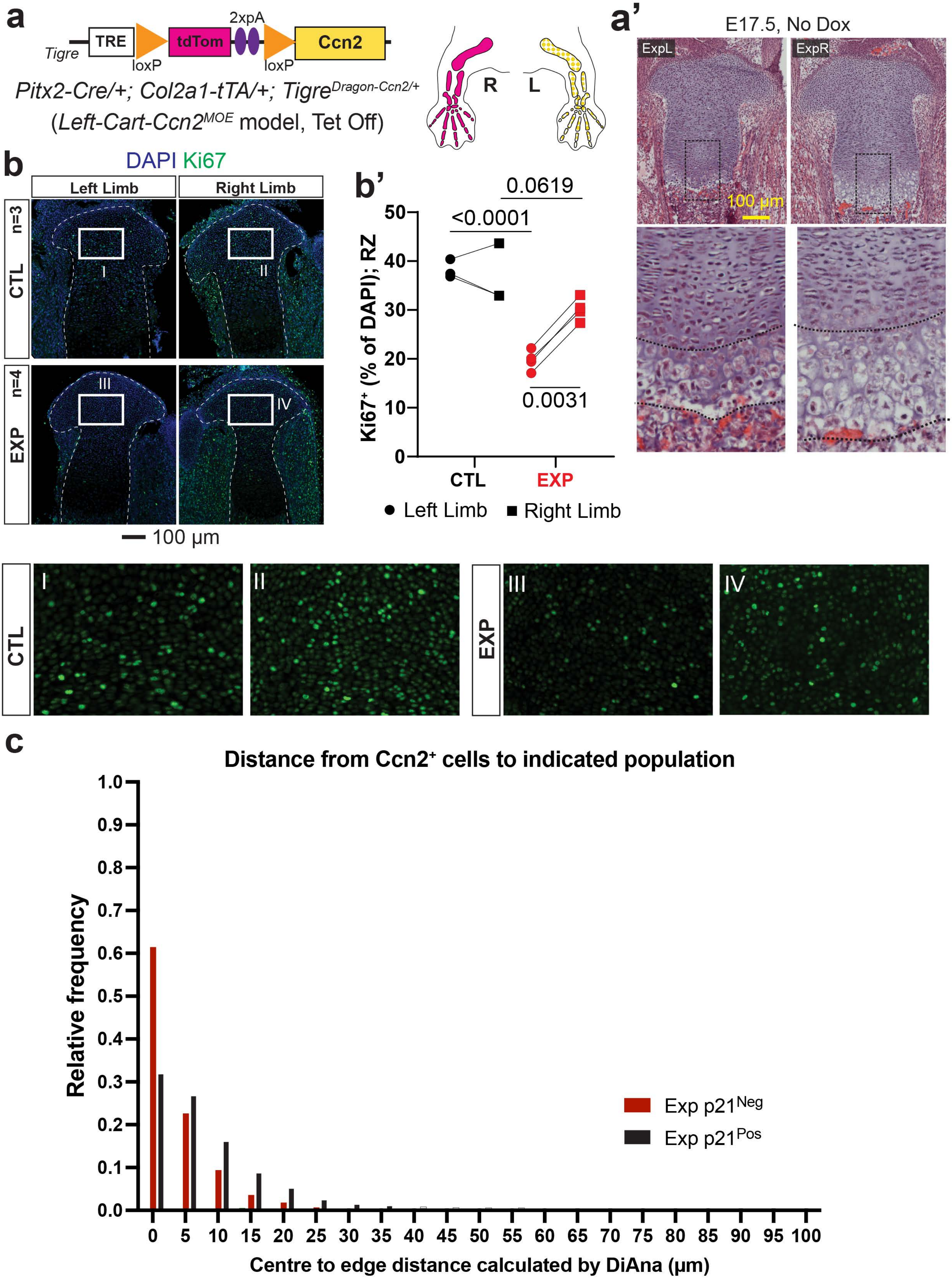
a
Tigre
TRE
loxP
tdTom
2xpA
loxP
Ccn2
Pitx2-Cre/+; Col2a1-tTA/+; Tigre^Dragon-Ccn2/+
(Left-Cart-Ccn2^MOE model, Tet Off)
R
L
a'
E17.5, No Dox
ExpL
ExpR
100 µm
b
DAPI Ki67
Left Limb
Right Limb
CTL
n=3
EXP
n=4
I
II
III
IV
100 µm
b'
Ki67⁺ (% of DAPI); RZ
0.0619
<0.0001
0.0031
CTL
EXP
Left Limb
Right Limb
c
Distance from Ccn2⁺ cells to indicated population
Relative frequency
Exp p21^Neg
Exp p21^Pos
Centre to edge distance calculated by DiAna (µm)

Supplementary Figure 10. Effect of *Ccn2* overexpression in the left cartilage. **a**, **a'**, Mouse model of unilateral, cartilage-targeted, *Ccn2* misexpression (a) and representative H&E images (a') of left and right proximal tibiae at E17.5 (n=3). **b**, **b'**, Representative images (b, boxed regions shown magnified in I-IV) and quantification (b') of Ki67 staining in the RZ of CTL and EXP proximal tibia (n=5 and 3, respectively.)

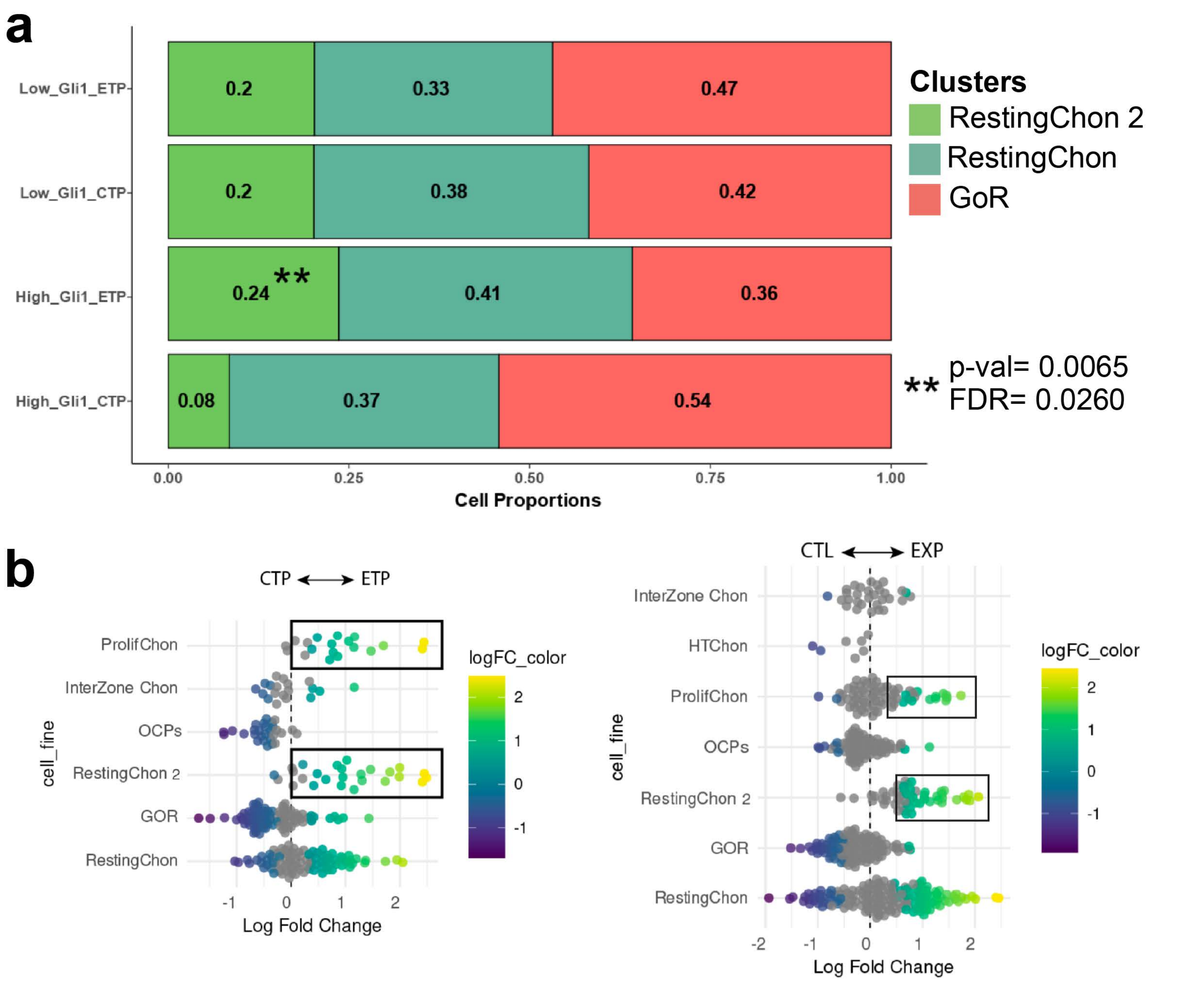

a
Low_Gli1_ETP
Low_Gli1_CTP
High_Gli1_ETP
High_Gli1_CTP
0.2
0.33
0.47
0.2
0.38
0.42
0.24**
0.41
0.36
0.08
0.37
0.54
0.00
0.25
0.50
0.75
1.00
Cell Proportions
Clusters
RestingChon 2
RestingChon
GoR
** p-val= 0.0065
FDR= 0.0260
b
CTP ⟷ ETP
ProlifChon
InterZone Chon
OCPs
RestingChon 2
GOR
RestingChon
cell_fine
Log Fold Change
logFC_color
CTL ⟷ EXP
InterZone Chon
HTChon
ProlifChon
OCPs
RestingChon 2
GOR
RestingChon
cell_fine
Log Fold Change
logFC_color

Supplementary Figure 11. Cell populations in experimental and control populations, belonging or not to the Gli1 lineage. **a**, Comparison of cell proportions between control (CTP) and *Pan-Cart-p21* (ETP) samples within the Gli1 lineage ($tdTom^{+}$), distinguishing between high- and low-Gli1 expression groups. GoR, groove of Ranvier. Note how the RestingChon 2 population is mainly expanded at the expense of the GoR one. *Propeller* was used for statistical comparison (p-values and FDR provided for the significant change). **b**, Similar comparison using *MiloR*[2]. Left: ETP vs. CTP (left). Right: merged ETP+ETN (EXP) vs. merged CTP+CTN (CTL). Each cell is given an enrichment score (logFC) in the experimental population. The rectangles delineate cell populations preferentially enriched in *Pan-Cart-p21* samples.

Supplementary Table 1

Gli1 (10g) module

ANOVA per cell type	p-value
OCPs	0 0446865
RestingChon	0 05522893
PHTChon	0 15615113
Dividing Chon	0 72610742
InterZone Chon	0 86095158
ProliferatingChon	0 95632759

multiple comparisons test (Sidak correction)	comparison	mean.diff	p.raw	p-adj
OCPs	ExpL-ExpR	0 01818012	0 02702733	0 07891031
RestingChon	Ctl-ExpL	-0 0083156	0 03526965	0 10212097
PHTChon	Ctl-ExpL	0 01888991	0 03757916	0 10855397
OCPs	Ctl-ExpL	-0 0143033	0 057882	0 10013491
RestingChon	ExpL-ExpR	0 00552006	0 15391047	0 394312
RestingChon	Ctl-ExpR	-0 0027956	0 42247521	0 80737533
Dividing Chon	Ctl-ExpL	0 00568663	0 42488968	0 80978118
PHTChon	Ctl-ExpR	0 00943431	0 46004991	0 84257965
PHTChon	ExpL-ExpR	-0 0094556	0 47266536	0 85335782
Dividing Chon	ExpL-ExpR	-0 0048883	0 47451957	0 85489926
InterZone Chon	ExpL-ExpR	0 00281005	0 58691943	0 92951377
OCPs	Ctl-ExpR	0 00387687	0 63148513	0 9499545
InterZone Chon	Ctl-ExpL	-0 0020655	0 67576366	0 96591329
ProliferatingChon	ExpL-ExpR	-0 0027538	0 77162692	0 98808937
ProliferatingChon	Ctl-ExpL	0 00169732	0 83167968	0 99523119
InterZone Chon	Ctl-ExpR	0 00074455	0 86507359	0 99754365
ProliferatingChon	Ctl-ExpR	-0 0010565	0 902976	0 99908665
Dividing Chon	Ctl-ExpR	0 00079836	0 90700497	0 99919577

Gli1_extended (13g) module

ANOVA per cell type	p-value
OCPs	0 01174893
RestingChon	0 05611665
PHTChon	0 14751049
Dividing Chon	0 5941093
InterZone Chon	0 74760945
ProliferatingChon	0 78162374

multiple comparisons test (Sidak correction)	comparison	mean.diff	p.raw	p-adj
OCPs	ExpL-ExpR	0 01847731	0 00732395	0 02181133
OCPs	Ctl-ExpL	-0 015275	0 02115584	0 06213429
RestingChon	Ctl-ExpL	-0 0069434	0 02473412	0 07238217
PHTChon	Ctl-ExpL	0 01472893	0 0338457	0 09813928
RestingChon	ExpL-ExpR	0 0051348	0 08831068	0 24222443
PHTChon	ExpL-ExpR	-0 0107181	0 28193988	0 62976078

Dividing Chon	Ctl-ExpL	0 00606065	0 30915998	0 67028974
Dividing Chon	ExpL-ExpR	-0 0052536	0 36067607	0 73868588
InterZone Chon	ExpL-ExpR	0 00309182	0 45610258	0 83910187
ProliferatingChon	Ctl-ExpL	0 00439925	0 48912201	0 86666273
RestingChon	Ctl-ExpR	-0 0018086	0 51239751	0 88406949
InterZone Chon	Ctl-ExpL	-0 0022936	0 56136654	0 91560723
OCPs	Ctl-ExpR	0 00320226	0 60637054	0 93900942
PHTChon	Ctl-ExpR	0 00401078	0 67440062	0 9654816
ProliferatingChon	ExpL-ExpR	-0 0030106	0 67640497	0 96611515
InterZone Chon	Ctl-ExpR	0 00079825	0 8169844	0 99386995
ProliferatingChon	Ctl-ExpR	0 0013887	0 8370141	0 99567038
Dividing Chon	Ctl-ExpR	0 00080709	0 8879384	0 99859275

Supplementary Table 1. Comparison of Gli1-related regulons across genotypes. ANOVA and multiple comparisons tests are shown.

Supplementary Data 1. Outcome of SCENIC analysis. For each genotype, population and Regulon, average expression, scaled expression (z-score) and percentage of expressing cells are indicated.

Supplementary References

1 Powell, D. R. Degust: interactive RNA-seq analysis,. https://doi.org:10.5281/zenodo.3258932

2 Dann, E., Henderson, N. C., Teichmann, S. A., Morgan, M. D. & Marioni, J. C. Differential abundance testing on single-cell data using k-nearest neighbor graphs. *Nature biotechnology* **40**, 245-253 (2022). https://doi.org:10.1038/s41587-021-01033-z
