# Supplementary material for: Gli1-expressing stromal cells are highly reparative precursors of long-lived chondroprogenitors in the fetal murine limb
Source: Nat Commun. 2025 Nov 18;16:10107. doi: 10.1038/s41467-025-65029-y (PMC12627582; doi:10.1038/s41467-025-65029-y)
Supplement: Supplementary file 2 — Description of Additional Supplementary Files [file 41467_2025_65029_MOESM2_ESM.pdf]

Description of Additional Supplementary Files

File Name: Supplementary Data 1
Description: Outcome of SCENIC analysis. For each genotype, population and Regulon, average expression, scaled expression (z-score) and percentage of expressing cells are indicated.

File Name: Supplementary Data 2
Description: Cell Profiler pipeline (with notes) to quantify individual and colocalised tdT, EdU and p21 signals from RGB images.
