# Supplementary material for: Gli1-expressing stromal cells are highly reparative precursors of long-lived chondroprogenitors in the fetal murine limb
Source: Nat Commun. 2025 Nov 18;16:10107. doi: 10.1038/s41467-025-65029-y (PMC12627582; doi:10.1038/s41467-025-65029-y)
Supplement: Supplementary file 5 — Reporting Summary [file 41467_2025_65029_MOESM5_ESM.pdf]

nature portfolio

Corresponding author(s): Rosello-Diez, Alberto

Last updated by author(s): Sep 11, 2025

Reporting Summary

Nature Portfolio wishes to improve the reproducibility of the work that we publish. This form provides structure for consistency and transparency in reporting. For further information on Nature Portfolio policies, see our Editorial Policies and the Editorial Policy Checklist.

Statistics

For all statistical analyses, confirm that the following items are present in the figure legend, table legend, main text, or Methods section.

n/a	Confirmed	
☐	☒	The exact sample size (*n*) for each experimental group/condition, given as a discrete number and unit of measurement
☐	☒	A statement on whether measurements were taken from distinct samples or whether the same sample was measured repeatedly
☐	☒	The statistical test(s) used AND whether they are one- or two-sided<br>*Only common tests should be described solely by name; describe more complex techniques in the Methods section.*
☒	☐	A description of all covariates tested
☐	☒	A description of any assumptions or corrections, such as tests of normality and adjustment for multiple comparisons
☐	☒	A full description of the statistical parameters including central tendency (e.g. means) or other basic estimates (e.g. regression coefficient) AND variation (e.g. standard deviation) or associated estimates of uncertainty (e.g. confidence intervals)
☐	☒	For null hypothesis testing, the test statistic (e.g. *F*, *t*, *r*) with confidence intervals, effect sizes, degrees of freedom and *P* value noted<br>*Give P values as exact values whenever suitable.*
☒	☐	For Bayesian analysis, information on the choice of priors and Markov chain Monte Carlo settings
☒	☐	For hierarchical and complex designs, identification of the appropriate level for tests and full reporting of outcomes
☒	☐	Estimates of effect sizes (e.g. Cohen's *d*, Pearson's *r*), indicating how they were calculated

Our web collection on statistics for biologists contains articles on many of the points above.

Software and code

Policy information about availability of computer code

Data collection	ZEN Blue and ZEN Black for microscopy imaging. Siemens Inveon software for micro-CT acquisition
Data analysis	ImageJ 1.4 and CellProfiler 4.0 for image analysis. Excel 16.8 and Prism 10 for statistical analysis. Mimics 21 for micro-CT measurements. Seurat was used for single-cell/nuclei analysis. Mimics and the Basilisc pipeline (PMID: 34513850) were used to measure bone length from micro-CT images

For manuscripts utilizing custom algorithms or software that are central to the research but not yet described in published literature, software must be made available to editors and reviewers. We strongly encourage code deposition in a community repository (e.g. GitHub). See the Nature Portfolio guidelines for submitting code & software for further information.

Data

Policy information about availability of data

All manuscripts must include a data availability statement. This statement should provide the following information, where applicable:

- Accession codes, unique identifiers, or web links for publicly available datasets
- A description of any restrictions on data availability
- For clinical datasets or third party data, please ensure that the statement adheres to our policy

The single-nuclei and single-cell RNA-seq datasets have been deposited at the NCBI Sequence Read Archive (SRA) with accession numbers PRJNA1136579 and PRJNA1138445, respectively. These can be accessed via the Gene Expression Omnibus (GEO), with accession numbers GSE273540 and GSE273538, respectively

Research involving human participants, their data, or biological material

Policy information about studies with human participants or human data. See also policy information about sex, gender (identity/presentation), and sexual orientation and race, ethnicity and racism.

Reporting on sex and gender	N/A
Reporting on race, ethnicity, or other socially relevant groupings	N/A
Population characteristics	N/A
Recruitment	N/A
Ethics oversight	N/A

Note that full information on the approval of the study protocol must also be provided in the manuscript.

Field-specific reporting

Please select the one below that is the best fit for your research. If you are not sure, read the appropriate sections before making your selection.

☒ Life sciences ☐ Behavioural & social sciences ☐ Ecological, evolutionary & environmental sciences

For a reference copy of the document with all sections, see nature.com/documents/nr-reporting-summary-flat.pdf

Life sciences study design

All studies must disclose on these points even when the disclosure is negative.

Sample size	G*Power was used to estimate sample sizes (5% significance level, 90% power), based on the variability observed in pilot studies.
Data exclusions	In experiments that require analyses of multiple tissue sections per sample, occasional sections were technically flawed (artifacts, damage) and had to be discarded from the quantifications, based on the Grubbs test. These are highlighted in red in the 'raw data' file
Replication	Image analysis was repeated at slightly different thresholds to ensure robustness of the analysis.
Randomization	No specific selection criteria was used to assign CTGF treatment to ex-vivo cultured bones, other than being consistent within the same experiment (i.e., in some experiments, left limbs were vehicle-treated and right limbs CTGF-treated, and in other experiments it was the opposite)
Blinding	The investigator performing image analysis (bone length, cell quantification on Cell Profiler) was blinded to the genotype

Reporting for specific materials, systems and methods

We require information from authors about some types of materials, experimental systems and methods used in many studies. Here, indicate whether each material, system or method listed is relevant to your study. If you are not sure if a list item applies to your research, read the appropriate section before selecting a response.

Materials & experimental systems

n/a	Involved in the study	
☐	☒	Antibodies
☐	☐	Eukaryotic cell lines
☐	☐	Palaeontology and archaeology
☐	☒	Animals and other organisms
☐	☐	Clinical data
☐	☐	Dual use research of concern
☐	☐	Plants

Methods

n/a	Involved in the study	
☒	☐	ChIP-seq
☒	☐	Flow cytometry
☒	☐	MRI-based neuroimaging

Antibodies

Antibodies used	mCherry (goat polyclonal, Origene Technologies #AB0040-200, diluted 1/500),<br>BrdU/CldU (rat monoclonal BU1/75 (ICR1), Abcam #ab6326 diluted 1/500,

GFP (chicken polyclonal, Abcam #ab13970, dilute 1/1000),
GFP (alpaca monoclonal, Chromotek #gb2AF488-50, 1/300),
Ki67 (rat monoclonal (SolA15), ThermoFisher #14-5698-82 diluted 1/100),
mKate2 (camel monoclonal 1H7, Abnova #RAB00798, diluted 1/100),
PDGFRA (goat polyclonal, R&D # AF1062, diluted 1/100)

Validation

Anti mCherry (note from supplier): In 293HEK cells transfected with CDS plasmid detects a band of 29 kDa by Western blot. This antibody (AB0040) recognizes very well tdTomato and does not recognize GFP.

Anti BrdU/CldU: This antibody reacts with BrdU in single stranded DNA, BrdU attached to a protein carrier or free BrdU. It detects nucleated cells in S-Phase which have had BrdU incorporated into their DNA. Also reacts with chlorodeoxyuridine but with reduced staining. The antibody does not react with thymidine. It has been reported in the literature that this antibody clone cross-reacts with EdU (PMID: 23272138) and some customers reported that it cross reacts with IdU.

Anti-GFP antibody (chicken): chicken polyclonal antibody and is validated for use in ICC/IF and WB. It was first used in a scientific publication in 2005 and has been cited over 3610 times in peer reviewed journals. Anti-GFP antibody (ab13970) has been validated in ICC/IF and WB. Anti-GFP antibody (ab13970) specifically detects GFP (UniProt ID: P42212; Molecular weight: 27kDa).

Anti-GFP nanobody: The GFP-Booster stabilizes, enhances, and reactivates the signal of GFP-fusion proteins. Due to its small size, the GFP-Booster enables higher image quality in epifluorescence, confocal, and super-resolution microscopy:
• Considerably higher tissue penetration rates
• Superior accessibility and labelling of epitopes in crowded cellular/organelle environments
• Less than 2 nm epitope-label displacement minimizes linkage error
• Monovalent VHHs do not cluster their epitopes
• Validation: structure and function characterized
• Consistent and reliable performance due to recombinant production

Anti ki67: SolA15 recognizes mouse and rat Ki-67, a 300 kDa nuclear protein. Ki-67 is present during all active phases of the cell cycle (G1, S, G2, and mitosis), but is absent from resting cells (G0). Ki-67 is detected within the nucleus during interphase but redistributes to the chromosomes during mitosis. Ki-67 is used as a marker for determining the growth fraction of a given population of cells. The SolA15 antibody also recognizes human, non-human primate and canine Ki-67. Applications Reported: This SolA15 antibody has been reported for use in immunohistochemical staining of formalin-fixed paraffin embedded (FFPE) tissue sections and frozen (paraformaldehyde fixed) sections and immunocytochemistry (ICC). Applications Tested: This SolA15 antibody has been tested by immunohistochemistry.

Anti mKate2: Recognizes TagRFP, Tag BFP and mTagBFP2.

Anti PDGFRA: Detects mouse and rat PDGF R alpha in direct ELISAs and Western blots. In direct ELISAs, less than 1% cross-reactivity with recombinant human (rh) PDGF R alpha, rhPDGF R beta, and recombinant mouse PDGF R beta is observed.

Eukaryotic cell lines

Policy information about cell lines and Sex and Gender in Research

Cell line source(s)

State the source of each cell line used and the sex of all primary cell lines and cells derived from human participants or vertebrate models.

Authentication

Describe the authentication procedures for each cell line used OR declare that none of the cell lines used were authenticated.

Mycoplasma contamination

Confirm that all cell lines tested negative for mycoplasma contamination OR describe the results of the testing for mycoplasma contamination OR declare that the cell lines were not tested for mycoplasma contamination.

Commonly misidentified lines
(See ICLAC register)

Name any commonly misidentified cell lines used in the study and provide a rationale for their use.

Palaeontology and Archaeology

Specimen provenance

Provide provenance information for specimens and describe permits that were obtained for the work (including the name of the issuing authority, the date of issue, and any identifying information). Permits should encompass collection and, where applicable, export.

Specimen deposition

Indicate where the specimens have been deposited to permit free access by other researchers.

Dating methods

If new dates are provided, describe how they were obtained (e.g. collection, storage, sample pretreatment and measurement), where they were obtained (i.e. lab name), the calibration program and the protocol for quality assurance OR state that no new dates are provided.

☐ Tick this box to confirm that the raw and calibrated dates are available in the paper or in Supplementary Information.

Ethics oversight

Identify the organization(s) that approved or provided guidance on the study protocol, OR state that no ethical approval or guidance was required and explain why not.

Note that full information on the approval of the study protocol must also be provided in the manuscript.

Animals and other research organisms

Policy information about studies involving animals; ARRIVE guidelines recommended for reporting animal research, and Sex and Gender in Research

Laboratory animals	Mus musculus. See methods fro GA strains
Wild animals	n/a
Reporting on sex	Sex was determined after tissue collection. Both sexes were included in the analysis, as growth differences have not been described at early stages. In the few experiments that used adult mice, no significant differences were found between males and females.
Field-collected samples	n/a
Ethics oversight	Monash Animal Ethics Committee (protocol #32263)<br>Cambridge-based project licence PP6324596

Note that full information on the approval of the study protocol must also be provided in the manuscript.

Clinical data

Policy information about clinical studies
All manuscripts should comply with the ICMJE guidelines for publication of clinical research and a completed CONSORT checklist must be included with all submissions.

Clinical trial registration	*Provide the trial registration number from ClinicalTrials.gov or an equivalent agency.*
Study protocol	*Note where the full trial protocol can be accessed OR if not available, explain why.*
Data collection	*Describe the settings and locales of data collection, noting the time periods of recruitment and data collection.*
Outcomes	*Describe how you pre-defined primary and secondary outcome measures and how you assessed these measures.*

Dual use research of concern

Policy information about dual use research of concern

Hazards

Could the accidental, deliberate or reckless misuse of agents or technologies generated in the work, or the application of information presented in the manuscript, pose a threat to:

No	Yes	
☒	☐	Public health
☒	☐	National security
☒	☐	Crops and/or livestock
☒	☐	Ecosystems
☒	☐	Any other significant area

Experiments of concern

Does the work involve any of these experiments of concern:

No	Yes	
☒	☐	Demonstrate how to render a vaccine ineffective
☒	☐	Confer resistance to therapeutically useful antibiotics or antiviral agents
☒	☐	Enhance the virulence of a pathogen or render a nonpathogen virulent
☒	☐	Increase transmissibility of a pathogen
☒	☐	Alter the host range of a pathogen
☒	☐	Enable evasion of diagnostic/detection modalities
☒	☐	Enable the weaponization of a biological agent or toxin
☒	☐	Any other potentially harmful combination of experiments and agents

Plants

Seed stocks

Report on the source of all seed stocks or other plant material used. If applicable, state the seed stock centre and catalogue number. If plant specimens were collected from the field, describe the collection location, date and sampling procedures.

Novel plant genotypes

Describe the methods by which all novel plant genotypes were produced. This includes those generated by transgenic approaches, gene editing, chemical/radiation-based mutagenesis and hybridization. For transgenic lines, describe the transformation method, the number of independent lines analyzed and the generation upon which experiments were performed. For gene-edited lines, describe the editor used, the endogenous sequence targeted for editing, the targeting guide RNA sequence (if applicable) and how the editor was applied.

Authentication

Describe any authentication procedures for each seed stock used or novel genotype generated. Describe any experiments used to assess the effect of a mutation and, where applicable, how potential secondary effects (e.g. second site T-DNA insertions, mosiacism, off-target gene editing) were examined.
